# Integrated care for older people and program evaluation

**DOI:** 10.3389/fpubh.2022.1036628

**Published:** 2022-10-18

**Authors:** Samuel Eloy Gutiérrez-Barreto, Juan Pablo Gutiérrez

**Affiliations:** ^1^Master's and Doctorate Programs in Medical, Dental, and Health Sciences, National Autonomous University of Mexico, Mexico City, Mexico; ^2^Center for Policy, Population and Health Research, National Autonomous University of Mexico, Mexico City, Mexico

**Keywords:** program evaluation, ICOPE, WHO, program theory, design evaluation, implementation

Healthcare systems worldwide are challenged by the increasingly aging populations, characterized by multimorbidity and declining physical and mental function (1). The capacity of health systems to adapt and transform to provide services to this population group has proven heterogeneous across countries. In Low and Middle-Income Countries (LMIC), health systems are challenged by constrained resources and health services organizations based on pre-transition conditions, that is, still designed with a focus on infectious diseases, so struggling to provide adequate and timely care (2, 3). As a part of the response to this challenge, there is a need for a specific healthcare model that suits older population care. The World Health Organization (WHO) developed such a model with the Integrated Care for Older People (ICOPE) approach that uses available evidence to provide countries with micro, meso, and macro implementation actions to guide the services and systems (4). The ICOPE program is a community-based approach that aims to strengthen health services by building person-centered long-term care systems with a coordinated model of care (4). ICOPE had three significant steps (a) know who the older people in the community in need of care are, (b) with a scorecard to assess the capacity of the services and systems to support integrated care, and (c) draft the implementation plan. For the final step, we argue that the ICOPE approach to fulfill its promise, as with any new intervention for public services, requires an in-country assessment of program theory and design to strengthen and guide the local implementation.

As an approach developed with a global perspective, the 19 implementation actions of ICOPE were developed to fit all countries. Still, its current implementation faces several barriers of heterogeneous magnitude overall, particularly in LMIC. For example, the cultural systems modulate access to health services in different ways, such as negative (discrimination, racism) or positive (strong support networks) (5). So when implementing and evaluating interventions aiming to increase access, a deep understanding and consideration of the local culture related to health are imperative (6). From the start, pre-existent constraints and a lack of focus on older populations imply limited infrastructure, personnel, and information systems for continuity of care (7). One way to identify the specific barriers and magnitudes in a particular country is through the analysis and refinement of the ICOPE theory of change (ToC), that is, the explicit model of how the model works that allows to identify why the program does what it says it does and, if it does, how it achieves the desired results (8). The general ToC derived from ICOPE documentation may be refined and adapted to reflect the specificities of the context and how this may affect the likelihood of successful implementation.

The approach of the ToC can articulate the rationale of the intervention from the expected or desired outcome back to the resources needed to accomplish it, including the identification of the steps (pre-conditions) required, key players, and assumptions involved. The evaluation of the proposed model requires an understanding of the context, answering questions such as if the goal and objectives of the ICOPE program are logical and plausible. These goals and objectives are feasible? Are the procedures for identifying members of the target population, delivering service to them, and sustaining that service through competition well-defined and sufficient? The process to answer these questions may use a consensus approach with relevant stakeholders. The ToC is graphically represented in a flowchart that integrates all the elements from the program's theory and makes them explicit. It contains the outcomes, indicators, assumptions, rationales, and linkages between the previous factors with a proper methodology described elsewhere (9). Examples of the use of ToC for evaluating public health interventions include the INSPIRE project, which used a ToC to provide a well-defined theory and design in a public healthcare program (10).

Once a context-specific ToC has been developed, a formal evaluation of the ICOPE design may improve the likelihood of success. While from the development of the ToC, it is expected that there is a strong base on the rationale of the ICOPE as the model is based on available evidence, discussing its assumptions and its implementation feasibility is given existing constraints, both in resources and in a managerial will for transformation. The ToC framework to define a plausible plan to evaluate the theory and design for ICOPE in LMIC has the advantage of addressing the public problem. It also requires a description of the expected changes in the participants' behaviors, attitudes, and skills. To define it, we need to review the ICOPE documents, interview the program stakeholders, and conduct site visits and observations of the program functions and circumstances.

Developing health interventions based on the best available evidence is a high resource-demanding process that multilateral and international organizations may facilitate (11). To better take advantage of these global public goods in the form of shared knowledge, the adoption of proposed models and interventions requires a formal process that includes an understanding of the logic behind the intervention –the ToC—and evaluation of that logic –the design—in the context where is expected to be implemented.

## Author contributions

All authors listed have made a substantial, direct, and intellectual contribution to the work and approved it for publication.

## Conflict of interest

The authors declare that the research was conducted in the absence of any commercial or financial relationships that could be construed as a potential conflict of interest.

## Publisher's note

All claims expressed in this article are solely those of the authors and do not necessarily represent those of their affiliated organizations, or those of the publisher, the editors and the reviewers. Any product that may be evaluated in this article, or claim that may be made by its manufacturer, is not guaranteed or endorsed by the publisher.
